# Reduction of experimental diabetic vascular leakage by delivery of angiostatin with a recombinant adeno-associated virus vector

**Published:** 2007-01-31

**Authors:** Mong-Ping Shyong, Fenq-Lih Lee, Ping-Chang Kuo, Ai-Ching Wu, Huey-Chung Cheng, Show-Li Chen, Tao-Hsin Tung, Yeou-Ping Tsao

**Affiliations:** 1 Institute of Clinical Medicine, National Yang-Ming University, Taipei, Taiwan; 2 Su-Ao Veterans Hospital, I-Lan, Taiwan; 3 Department of Ophthalmology, Taipei Veterans General Hospital, Taipei, Taiwan; 4 Department of Medical Research, Mackay Memorial Hospital, Taipei, Taiwan; 5 Department of Ophthalmology, Mackay Memorial Hospital, Taipei, Taiwan; 6 Department of Microbiology, School of Medicine, National Taiwan University, Taipei, Taiwan; 7 Department of Medicine Research and Education, Cheng Hsin Rehabilitation Medical Center, Taipei, Taiwan; 8 Department of Microbiology and Immunology, National Defense Medical Center, Taipei, Taiwan

## Abstract

**Purpose:**

To evaluate the efficacy of recombinant adeno-associated virus (rAAV) vector expressing mouse angiostatin (Kringle domains 1 to 4) in reducing retinal vascular leakage in an experimental diabetic rat model.

**Methods:**

rAAV-angiostatin was delivered by intravitreal injection to the right eyes of Sprague-Dawley rats. As a control, the contralateral eye received an intravitreal injection of rAAV-lacZ. Gene delivery was confirmed by reverse-transcriptase polymerase chain reaction (RT-PCR). Diabetes was induced by intravenous injection of streptozotocin (STZ). Vascular permeability changes were evaluated by extravascular albumin accumulation and leakage of intravenous-injected fluorescein isothiocynate-bovine serum albumin (FITC-BSA). Effects of rAAV-angiostatin on expression of vascular endothelial growth factor (VEGF), pigment epithelium-derived factor (PEDF), occludin, and phospho-p42/p44 MAP kinase in retina tissue were analyzed by western blotting.

**Results:**

The rAAV-angiostatin injections led to sustained angiostatin gene expression in retina as confirmed by RT-PCR, and reduced extravascular albumin accumulation in STZ-induced diabetic retina. Further, rAAV-angiostatin significantly decreased intravascularly injected FITC-BSA leakage at 5 days (p=0.001), 10 days (p<0.001), and 15 days (p=0.001) after STZ-induced diabetes, as compared to the control eyes receiving rAAV-lacZ. Expression of VEGF and phosphorylation of p42/p44 MAP kinase in retina was reduced by rAAV-angiostatin at day 1 (p=0.043 for both VEGF and phospho-p42/p44 MAP kinase) after STZ-induced diabetes compared with rAAV-lacZ eyes. rAAV-angiostatin reduced retinal occludin loss at 10 days after STZ-induced diabetes (n=5, p=0.041). There was no significant difference in retinal PEDF expression between eyes injected with rAAV-angiostatin and rAAV-lacZ.

**Conclusions:**

Intravitreal delivery of rAAV-angiostatin reduces vascular leakage in an STZ-induced diabetic model. This effect is associated with a reduction in the retinal occludin loss induced by diabetes and downregulation of retinal VEGF and phosphor-p42/p44 MAP kinase expression. This gene transfer approach may reduce diabetic macular edema, providing protection in diabetic patients at risk for macular edema.

## Introduction

Diabetes mellitus is the most prevalent endocrine disease in developed countries [1], and diabetic retinopathy is the leading cause of blindness in the world [2,3]. Blood-retinal barrier (BRB) breakdown, increased vascular permeability and vascular leakage are early complications of diabetes and a major cause of diabetic macular edema [4-6]. As there is no satisfactory or noninvasive therapy, diabetic macular edema is a major cause of vision loss in diabetic patients [7].

An ideal treatment strategy would be to deliver a therapeutic gene with a vector that could confer long-term transgene expression and tissue protection with a single administration. We have previously reported that a recombinant adeno-associated virus vector expressing angiostatin (rAAV-angiostatin) suppressed laser-induced choroidal neovascularization [8]. Recently, an effect of angiostatin in reducing vascular permeability in the retina in diabetic and oxygen-induced retinopathy models was reported [9].

Angiostatin (Kringles 1 through 4) is a proteolytic fragment of plasminogen [10]. It was identified as a potent angiogenic inhibitor, which blocks neovascularization and suppresses tumor growth and metastases [10,11]. Angiostatin specifically inhibits proliferation, induces apoptosis in vascular endothelial cells [12], and downregulates vascular endothelial growth factor (VEGF), the latter via inactivation of the p42/p44 MAP kinase pathway [9,13,14]. We also noted that some proteolytic fragments of plasminogen can induce upregulation of pigment epithelium-derived factor (PEDF) expression, a potent angiogenic inhibitor in experimental diabetes [15].

BRB breakdown may be due to disassembly of unique proteins that constitute the functional vascular endothelial tight junction [16-18]. VEGF is a potent angiogenic factor [13], whose overproduction in the retina has been noted in the development of vascular hyperpermeability in diabetes [14]. Furthermore, VEGF affects the tight junction protein occludin, inducing occludin phosphorylation [19] and redistribution [20]; resultant occludin reduction is associated with BRB breakdown in diabetes [21].

The present study was designed to examine the transgenic expression of rAAV-angiostatin in the eye and its effect on vascular permeability in the streptozotocin (STZ)-induced diabetic model. Since it has been demonstrated angiostatin can induce the downregulation of VEGF through the blockade of phosphorylation of p42/p44 MAP kinase [9,13,14], we also studied the relationship between rAAV-angiostatin, p42/p44 MAP kinase, occludin, VEGF, and PEDF in this model.

## Methods

### Animals

Male Sprague-Dawley (SD) rats (Charles River Laboratories, WilmingtonMA) weighing approximately 200 g on arrival were used in this study. The animals were cared for in accordance with the ARVO Statement for the Use of Animals in Ophthalmic and Vision Research. All experimental procedures used aseptic sterile techniques and were approved by the Animal Care and Use Committee of the Mackay Memorial Hospital.

### Generation of rAAV-angiostatin

cDNA coding for angiostatin was amplified by polymerase chain reaction (PCR) according to a published report [22]. rAAV encoding mouse angiostatin cDNA or lacZ were constructed by using a three-plasmid cotransfection system as described previously [22-24]. Titers of rAAV-angiostatin and rAAV-lacZ were determined by dot blot hybridization using angiostatin cDNA and lacZ as probes [25].

### Intravitreal injections of rAAV-angiostatin

After being anesthetized, each animal received an intravitreal injection of rAAV- angiostatin (5 μl, 1.5x10^10^ viral particles) as described previously [26]. The contralateral eye of each rat was injected with rAAV-lacZ to serve as a control.

### Experimental Diabetes

Experimental diabetes was induced three weeks after intravitreal injection of rAAV. Diabetes was induced with a single 60 mg/kg intravenous injection of streptozotocin (Sigma-Aldrich, St. Louis, MO) in 10 mM citrate buffer, pH 4.5. Animals that served as nondiabetic controls received an equivalent amount of citrate buffer alone [21] Twenty-four h later, rats with blood glucose levels higher than 250 mg/dl were deemed diabetic. These diabetic rats received 6-8U NPH insulin (Lilly, Indianapolis, IN) once a week to prevent ketoacidosis. Just before experimentation, blood glucose levels were measured again to confirm diabetic status.

### Reverse transcription-polymerase chain reaction

Expression of rAAV-angiostatin in retina was confirmed by RT-PCR according to a protocol described in reference [8]. Each rat eye that was previously injected with rAAV-angiostatin and rAAV-lacZ was enucleated, and chorioretinal tissues were harvested for RT-PCR at 1, 5, 10, and 15 days after induction of experimental diabetes. The cDNA was synthesized using oligo(dT) primer and 200 IU transcriptase (SuperScript II; Life Technologies, Carlsbad, CA) according to the manufacturer's instructions. PCR amplification was performed with two oligonucleotide primers, 5'-CAG CAA TGC GTG ATC ATG-3' and 5'-TGG AGA TTT TGC CCT CAT AC-3'. As a control, PCR amplification was performed for glyceradehyde-3-phosphate dehydrogenase (GAPDH) with two oligonucleotide primers, 5'-GGA AGG GCT CAT GAC CAC AG-3' and 5'-CCT TTA GTG GGC CCT CGG-3'. To rule out the possibility that gene amplification products were derived from amplification of contaminating angiostatin genomic DNA, we treated the total RNA with RNase free DNase I (Qiagen, Valencia, CA) before RT-PCR.

### Immunofluorescence assay

Five-μm-thick retinal tissue sections from formalin-fixed, paraffin-embedded blocks were transferred to positively charged slides to be used for staining. Sections were dewaxed in xylene and progressively hydrated [27]. They were then washed three times with PBS and a 1:400 dilution of polyclonal rabbit antihuman albumin antibody (DAKO Diagnostics. Mississauga, ON, Canada) was applied. A fluorescein isothiocyanate (FITC)-conjugated antimouse IgG was used as a secondary antibody. The results were viewed with a fluorescence microscope (Zeiss Axioplan HBO100, Oberkochen, Germany).

### Measurement of leakage with intravascular injected FITC-BSA

Retinal vascular leakage was measured using the intravascular injected FITC-BSA as previously described [21,28] with some modifications. After induction of anesthesia, the rats received tail vein injections of 100 mg/kg FITC-bovine serum albumin (FITC-BSA, Sigma-Aldrich). The animals were sacrificed 20 min later, and their eyes were removed, embedded in OCT medium, and snap-frozen in liquid nitrogen. The plasma was collected and assayed for fluorescence with an SPEX fluorescence spectrophotometer (Molecular Devices, Sunnyvale, CA) based on standard curves of FITC-BSA in normal rat plasma. Frozen retinal sections (6 μm thick) collected every 60 μm were viewed with a Zeiss Axioplan HBO100 fluorescence microscope. Images from six retinal nonvascular areas (200 μm^2^) in each section were collected. Quantification of FITC-BSA fluorescence intensity was calculated by computer software Q-win (Leica, Wetzlar, Germany) and normalized to plasma fluorescence intensity for each animal.

### Western blot analysis of VEGF, PEDF, occludin, and p42/p44 MAP kinase

Rats were sacrificed, and their eyes were removed for western blotting analysis at 1, 5, 10, and 15 days after STZ treatment. The chorioretinal tissue was harvested, and the soluble fractions were prepared by homogenizing the retina in Eppendorf tubes containing RIPA lysis buffer (50 mM Tris, 150 mM NaCl, 10 mM EDTA, 0.1% SDS, 1% NP-40, 0.5% sodium deoxycholate, 1 mM Na_3_VO_4_, 1 mM NaF, 1 mM EGTA, 1 mM PMSF, 1 mg/ml leupeptin, and 1 mg/ml pepstatin A). Proteins (50 μg) were extracted for electrophoresis on 10% SDS-polyacrylamide gels. The membranes were incubated with antibody specific to VEGF [29,30], PEDF [31], p42/p44 MAP kinase [21] (all from Santa Cruz Biotechnology, Inc., Santa Cruz, CA) and occludin [20] (Zymed, San Francisco, CA). The results were semiquantified by densitometry (Fujifilm LAS3000, Tokyo, Japan) and normalized to actin levels.

### Cell culture and rAAV-angiostatin infection

To further evaluate whether angiostatin could induce the expression of PEDF in endothelial cells, we infected human umbilical vein endothelial cells (HUVEC-2C, Cascade Biologics, Portland, OR) with rAAV-angiostatin. The HUVEC-2C cells were cultured in medium 200 (Cascade Biologics) containing low serum growth supplement. All cells were supplemented with 1% penicillin-streptomycin and maintained at 37 °C and 5% CO_2_. Confluent cells obtained during the fifth passage were used for rAAV-angiostatin infection. The HUVEC-2C cells were infected by rAAV-angiostatin in Dulbeco's modified Eagle's medium for two days. Cell lysates were then prepared and analyzed for PEDF by western blotting as described in the previous paragraph.

### Statistical Analyses

The results are expressed as the mean±SD. Retinal FITC-BSA fluorescence intensity was analyzed in serial retinal sections from four rats at 5, 10, and 15 days after induction of diabetes. Due to skewed distributions, data were subjected to logarithmic transformation for analysis. Differences of retinal FITC-BSA fluorescence intensity between eyes receiving rAAV-angiostatin and rAAV-lacZ injection at 5, 10, and 15 days after induction of diabetes were analyzed by a paired-sample Student's t test. The retinal expression of VEGF, PEDF, phosporylation of p42/p44 MAPK, and occludin were analyzed by the Wilcoxon signed-rank test. All p-values are two-tailed, and differences were considered to be statistically significant for p<0.05.

## Results

### Gene delivery by rAAV-angiostatin

**Figure 1:**
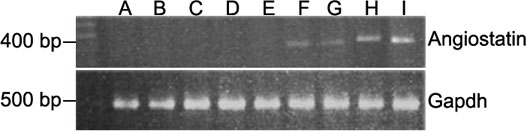
RT-PCR analysis of angiostatin cDNA in chorioretinal tissue. The eyes previously injected with rAAV-lacZ (lanes B to E) and rAAV-angiostatin (lanes F to I) were enucleated and chorioretinal tissues were harvested for RT-PCR at 1, 5, 10, and 15 days after induction of experimental diabetes. Lane A is the control eye. Lanes B and F are 1 day after diabetes induction. Lanes C and G are 5 days after diabetes induction. Lanes D and H are 10 days after diabetes induction. Lanes E and I are 15 days after diabetes induction. There was no angiostatin gene expression in the control eye (lane A) and eyes injected with rAAV-lacZ (lanes B to E). In the eyes injected with rAAV-angiostatin, angiostatin gene expression was detected (lanes F to I). As an internal control, expression of GAPDH was detected in normal control eye and eyes receiving both rAAV-angiostatin and rAAV-lacZ injections (lanes A to I). "M" indicates molecular weight markers.

There was no angiostatin gene expression in normal control eyes (Figure 1, lane A) and eyes injected with rAAV-lacZ at 1, 5, 10, and 15 days after induction of diabetes (Figure 1, lanes B-E). In the eyes injected with rAAV-angiostatin, angiostatin gene expression was detected at 1, 5, 10, and 15 days after induction of diabetes (Figure 1, lanes F-I). As an internal control, expression of GAPDH was detected in normal control eye and eyes receiving both rAAV-angiostatin and rAAV-lacZ injections (Figure 1, lanes A-I).

### Induction of experimental diabetes

Animals (n=12) with a blood glucose exceeding 250 mg/dl were selected for inclusion in the diabetic group. All diabetic animals had higher blood glucose levels and reduced body weight gain at 5, 10, and 15 days after induction of diabetes compared to age-matched, nondiabetic control animals (Table 1).

**Table 1:** Animal physiological variables.

			**After streptozotocin induction of diabetes**								
	**Baseline (n=4)**		**5 days (n=4**			**10 Days (n=4)**			**15 Days (n=4)**		
**sample**	**BW (g)**	**Blood sugar (mg/dl)**	**BW (g)**	**Increase in body weight (%)**	**Blood sugar (mg/dl)**	**BW (g)**	**Increase in body weight (%)**	**Blood sugar (mg/dl)**	**BW (g)**	**Increase in body weight (%)**	**Blood sugar (mg/dl)**
Age-matched control rat	248+14	139+13	260+13	4.8	141+11	294+10	18.5	154+12	312+9	25.8	143+11
STZ-induced diabetic rat	254+11	143+9	257+10	2.4	352+15	270+14	7.7	377+13	280+11	11.6	396+15

Animals were made diabetic by a single streptozocin (STZ) injection (65 mg/kg) in 1 mmol/l sodium citrate buffer, pH 4.5. All diabetic animals had higher blood glucose levels and reduced body weight gain compared to age matched, non-diabetic control animals.

### rAAV-angiostatin influence on vascular permeability

**Figure 2:**
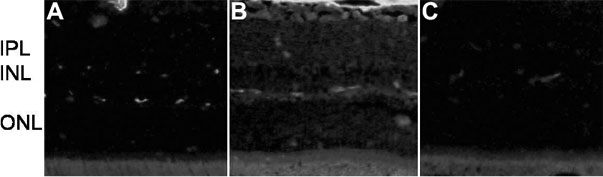
Representative retinal sections following immunostaining for albumin. Increased immunostaining was present throughout the retina one week after STZ-induction of diabetes in eyes receiving rAAV-lacZ injection (**B**) compared to the normal Sprague-Dawley rat (**A**) where staining was restircted to blood vessels. **C**: Intravitreal injection of rAAV-angiostatin decreased immunostaining in the retina one week after induction of diabetes. Magnification X200. IPL denotes the inner plexiform layer, INL indicates the inner nuclear layer, and ONL marks the outer nuclear layer.

One week after induction of diabetes, the immunofluorescence assay, using anti-albumin antibody, disclosed staining only inside blood vessels in normal control eyes (Figure 2A). Increased extravascular albumin in the retinal parenchyma was seen in the eyes of diabetic animals receiving rAAV-lacZ (Figure 2B). In the diabetic animals receiving rAAV- angiostatin, however, albumin staining was observed only inside blood vessels (Figure 2C).

**Figure 3:**
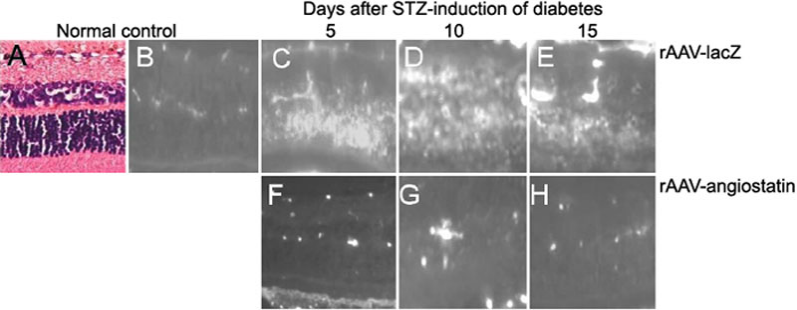
FITC-BSA fluorescence in normal and streptozotocin (STZ)-induced diabetic rat retina. **A**: Hematoxylin and eosin staining of control retina. **B**: FITC-BSA fluorescence is limited to the vasculature in the normal retina and in **C** is diffusely increased throughout the retinal parenchyma at 5 days after STZ-induced diabetes in eyes receiving rAAV-lacZ injection. Increased fluorescence intensity throughout the retinal parenchyma is still present at 10 (**D**) and 15 (**E**) days after STZ-induced diabetes in eyes receiving rAAV-lacZ injection. **F**: Little fluorescence was present in the retinal parenchyma in eyes receiving rAAV-angiostatin injection at 5 days after induction of diabetes. Retinal parenchyma fluorescence at 10 (**G**) and 15 (**H**) days after induction of diabetes in eyes with rAAV-angiostatin injection was decreased as compared with eyes with rAAV-lacZ injection. Original magnification was 200X.

To confirm the effect of rAAV-angiostatin on vascular permeability, we examined animals injected with intravenous FITC-BSA 5, 10, and 15 days after induction of diabetes. Figure 3 shows representative micrographs of the eyes of normal control and STZ-induced diabetic rats. FITC-BSA fluorescence is limited to the vasculature in the normal retina (Figure 3B) and diffusely increased throughout the retinal parenchyma at 5 days after STZ-induced diabetes in eyes receiving rAAV-lacZ injection (Figure 3C). Increased fluorescence intensity throughout the retinal parenchyma is still present at 10 (Figure 3D) and 15 (Figure 3E) days after STZ-induced diabetes in eyes receiving rAAV-lacZ injection. Little fluorescence was present in the retinal parenchyma in eyes receiving rAAV-angiostatin injection (Figure 3F) at 5 days after induction of diabetes. Retinal parenchyma fluorescence at 10 (Figure 3G) and 15 (Figure 3H) days after induction of diabetes in eyes with rAAV-angiostatin injection was decreased as compared with eyes that received the rAAV-lacZ injection.

**Figure 4:**
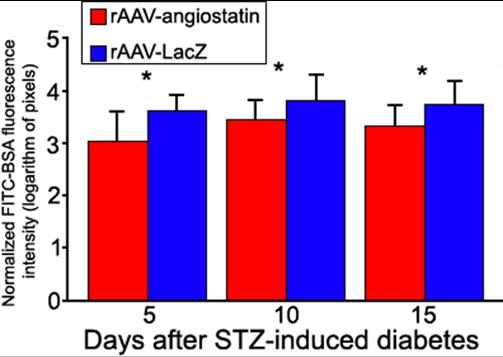
Quantification of vascular leakage in experimental diabetes. FITC-BSA fluorescence intensity was measured by image analysis in serial retinal sections. Rats each received an intravenous injection of FITC-BSA were sacrificed at 5, 10, and 15 days after induction of diabetes. The average retinal FITC-BSA fluorescence intensity was calculated and normalized to plasma fluorescence intensity. The retinal FITC-BSA fluorescence intensity in eyes receiving rAAV-angiostatin injection was 2.99±0.62 pixels at 5 days, 3.42±0.38 pixels at 10 days and 3.30±0.40 pixels at 15 days after induction of diabetes. The retinal FITC-BSA fluorescence intensity in eyes receiving rAAV-lacZ injection was 3.59±0.31 pixels at 5 days, 3.77±0.51 pixels at 10 days and 3.71±0.47 pixels at 15 days after induction of diabetes. The normalized FITC-BSA fluorescence intensity in eyes receiving rAAV-angiostatin was decreased as compared to eyes receiving rAAV-lacZ at 5 days (t=3.67, n=49, p=0.001), 10 days (t=3.94, n=51, p<0.001), and 15 days (t=3.52, n=56, p=0.001) after STZ-induction of diabetes. The asterisk indicates a p less than or equal to 0.001. Four SD rats were represented by the number of sections (n) to be examined.

The retinal FITC-BSA fluorescence intensity was calculated and normalized to plasma fluorescence intensity by image analysis of serial sections (Figure 4). Four SD rats were represented by the number of sections to be examined at each timepoint. The mean retinal FITC-BSA fluorescence intensity in eyes receiving rAAV-angiostatin injection was 2.99±0.62 pixels at 5 days, 3.42±0.38 pixels at 10 days, and 3.30±0.40 pixels at 15 days after induction of diabetes. The retinal FITC-BSA fluorescence intensity in eyes receiving rAAV-lacZ injection was 3.59±0.31 pixels at 5 days, 3.77±0.51 pixels at 10 days, and 3.71±0.47 pixels at 15 days after induction of diabetes. Quantitative analysis showed that the fluorescence was decreased in eyes receiving rAAV-angiostatin as compared to eyes receiving rAAV-lacZ at 5 days (t=3.67, n=49, p=0.001), 10 days (t=3.94, n=51, p<0.001), and 15 days (t=3.52, n=56, p=0.001) after induction of diabetes.

### rAAV-angiostatin influence on occludin loss

**Figure 5:**
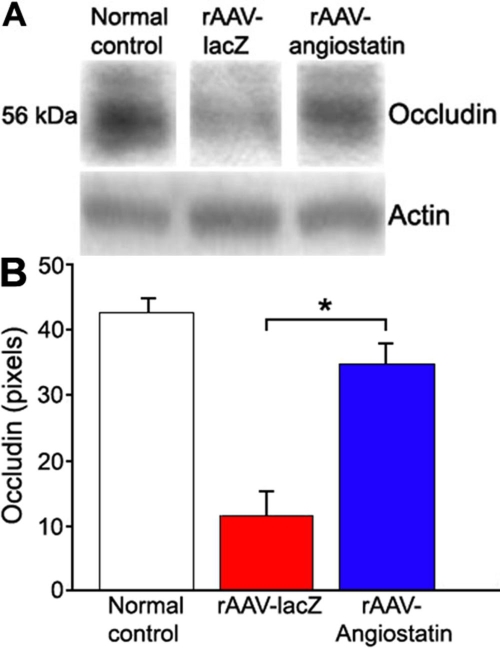
The effect of rAAV-angiostatin gene transfer on retinal occludin expression 10 days after STZ-induction of diabetes The rats received intravitreal injection of rAAV-angiostatin in the right eyes and rAAV-lacZ in left eyes, and diabetes was induced three weeks after injection. **A**: Each blot is a representative of the results from five rats. **B**: Occludin levels were semi-quantified by densitometry, and normalized to actin. The rAAV-angiostatin significantly decreased retinal occludin loss as compared to eyes receiving rAAV-lacZ injection at 10 days after induction of diabetes (the asterisk indicates significance using the Wilcoxon signed rank test, n=5, p=0.041).

Rats receiving rAAV-angiostatin in their right eyes and rAAV-lacZ in their left eyes were sacrificed, and their eyes were enucleated for western blotting analysis at 1, 5, 10, and 15 days after induction of diabetes. The retinal occludin content in normal control eye was 42.35±2.67 pixels. No differences in retinal occludin content were detected between eyes receiving rAAV-lacZ and rAAV-angiostatin injection at 1 (rAAV-lacZ: 45.82±3.08 pixels, rAAV-angiostatin: 43.62±2.78) and 5 days (rAAV-lacZ: 38.96±2.22 pixels, rAAV-angiostatin: 40.65±3.46 pixels) after induction of diabetes. Ten days after diabetes induction, retinal occludin content in eyes that received rAAV-lacZ was 11.35±3.57 pixels and 34.73±3.17 pixels in eyes that received rAAV-angiostatin occludin, a statistically significant reduction (n=5, p=0.041, Figure 5). The retinal occludin content in rAAV-lacZ-treted eyes was higher (26.32±3.46 pixels) at 15 days after induction of diabetes than it was at 10 days, and there was no difference in rAAV-angiostatin-treated animals (29.21±2.94 pixels).

### rAAV-angiostatin influence on VEGF expression

**Figure 6:**
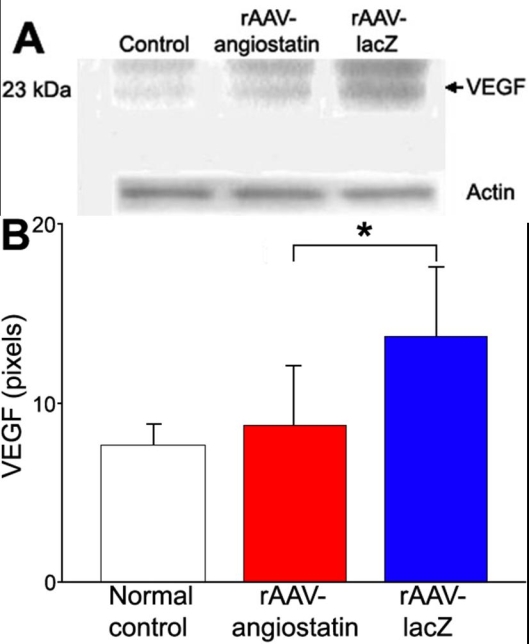
The effect of rAAV-angiostatin gene transfer on retinal VEGF expression at 1 day after STZ-induction of diabetes **A**: The blots show representative of results from six rats. **B**: VEGF levels were semiquantified by densitometry and normalized by actin levels. rAAV-angiostatin decreased the expression of VEGF as compared to eyes with rAAV-lacZ injection (the asterisk indicates significance using the Wilcoxon signed rank test, n=6, p=0.043).

One day after diabetes induction, we observed rAAV-angiostatin-mediated influence of retinal VEGF expression. The retinal VEGF expression in normal control SD rat was 7.56±1.25 pixels. Retinal VEGF was 13.7±3.78 pixels (n=6) in rAAV-lacZ-treated eyes and decreased to 8.69±3.23 pixels (n=6) in rAAV-angiostatin-treated eyes (Figure 6, p=0.043). The retinal VEGF expression in eyes receiving rAAV-lacZ was 11.45±2.73 pixels at 5 days, 9.12±3.12 pixels at 10 days, and 10.41±3.36 pixels at 15 days after induction of diabetes. The retinal VEGF expression in rAAV-angiostatin-treated eyes was 9.52±3.96 pixels at 5 days, 8.31±2.67 pixels at 10 days, and 10.16±3.36 pixels at 15 days after induction of diabetes. There was no statistical difference in retinal VEGF expression in rAAV-lacZ- and rAAV-angiostatin-treated eyes at 5, 10, and 15 days after induction of diabetes.

### PEDF expression

**Figure 7:**
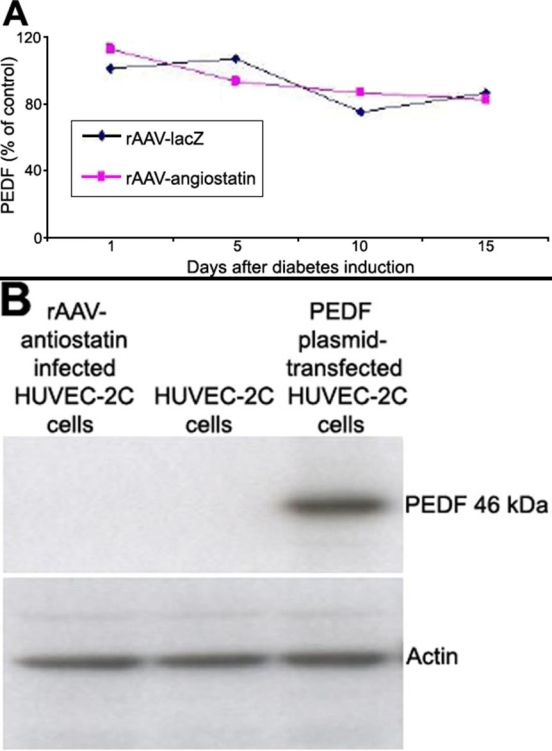
The effect of rAAV-angiostatin gene transfer on retinal PEDF expression **A**: There was no apparent difference in retinal PEDF expression between eyes exposed to rAAV-angiostatin and rAAV-lacZ. at 1, 5, 10, or 15 days after STZ injection. **B**: There was no increase in PEDF expression in HUVEC-2C cells receiving rAAVangiostatin compared to control HUVEC-2C cells. Western blotting of the PEDF plasmid-transfected HUVEC-2C cells was used as a positive control.

There was no significant difference in retinal PEDF expression between eyes exposed to rAAV-angiostatin and rAAV-lacZ at 1, 5, 10, or 15 days after STZ injection (Figure 7A). To further evaluate if rAAV-angiostatin could influence PEDF expression, we infected HUVEC-2C cells with rAAV-angiostatin. No PEDF expression was detected in rAAV-angiostatin-infected HUVEC-2C cells (Figure 7B). Western blot analysis of PEDF plasmid-transfected HUVEC-2C cells was used as a positive control.

### rAAV-angiostatin-mediated downregulation of phospho-p42/p44 MAP kinase

**Figure 8:**
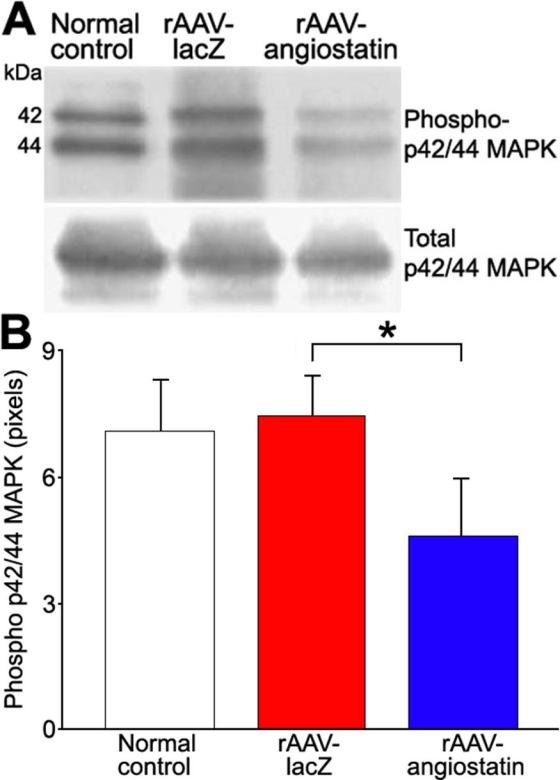
The effect of rAAV-angiostatin gene transfer on retinal phosphorylation of p42/p44 MAP kinases at 1 day after STZ-based induction of diabetes **A**: Representative blots show retinal phospho-p42/44 MAP kinase and total p42/44 MAP kinase in normal control eyes and in eyes injected with rAAV-lacZ or rAAV-angiostatin. **B**: Retinal levels of phosphor-p42/p44 MAP kinase one day after STZ-based induction of diabetes was reduced by rAAV-angiostatin (the asterisk indicates significance using the Wilcoxon signed rank test, n=5, p=0.043).

The retinal phosphor-p42/p44 MAP kinase level of normal control SD rat was 7.03±1.22 pixels. At one day after STZ injection, retinal phosphor-p42/p44 MAP kinase levels were 7.43±0.93 pixels (n=5) in eyes receiving rAAV-lacZ, and was decreased to 4.58±1.36 pixels(n=5) in eyes receiving rAAV-angiostatin (Figure 8, p=0.043). No effect was observed at 5 days after STZ injection (5.64±1.28 pixels for eyes receiving rAAV-lacZ, and 5.44±1.65 pixels for eyes receiving rAAV-angiostatin), 10 days (5.13±0.92 pixels for eyes receiving rAAV-lacZ, and 5.97±1.87 pixels for eyes receiving rAAV-angiostatin) and 15 days (7.37±1.33 pixels for eyes receiving rAAV-lacZ, and 7.25±2.65 pixels for eyes receiving rAAV-angiostatin)

## Discussion

Retinal vascular leakage is a major cause of macular edema in diabetic retinopathy and other retinal diseases [1-6]. Traditionally, laser photocoagulation has been used to reduce the vascular leakage induced by diabetes [32,33]. Recently, anti-inflammatory drugs such as triamcinolone [34], or pars-plana vitrectomy with or without internal limiting membrane peeling [35] have been used to attenuate the diabetic macular edema. Due to the duration and severity, an ideal strategy would be to develop an approach involving a single administration of a vector that would result in long-term expression of a suitable therapeutic gene [36].

rAAV vectors are highly efficient gene delivery systems which can facilitate long-term transduction [22,23]. We have previously reported in several animal models a gene transfer technique based on the use of rAAV vectors [8,23,25,26,37,38]. This gene transfer technique is particularly attractive for treating ocular disease for reasons of accessibility and long term transduction, and potentially because it would enable clinicians to avoid repeated intravitreal injection [8,37]. We previously reported suppression of laser-induced choroidal neovascularization by an rAAV vector expressing angiostatin [8]. Here, we report that vascular leakage in experimental diabetic rats can be reduced by angiostatin delivery via an rAAV vector. These results suggest that rAAV-angiostatin could be beneficial in the treatment of diabetic macular edema.

Angiostatin is a proteolytic fragment of plasminogen and contains kringle domains 1 through 4 [10]. It has been determined that angiostatin is a potent anti-angiogenic factor [11] that can inhibit endothelial cell migration and induce apoptosis in these cells [12]. Recently, intravitreal injection of angiostatin was found to reduce vascular leakage in a rat model of experimental diabetes and in oxygen induced retinopathy [9]. In the same report, the expression of VEGF was found to be downregulated by angiostatin.

VEGF is a potent angiogenic factor, expression of which is increased in eyes with diabetic retinopathy [13,14]. VEGF, which is also known as vascular permeability factor (VPF) [39], increases microvascular permeability at very low concentrations [39], and may be important in the pathogenesis of vascular leakage induced by diabetes [40]. Angiostatin-induced reduction of vascular leakage occurs through blockade of VEGF expression [15]. In our study, retinal VEGF expression decreased in eyes receiving rAAV-angiostatin as compared to rAAV-lacZ treated eyes at one and five days after induction of diabetes. Vascular leakage was also decreased in eyes receiving intravitreal injection of rAAV-angiostatin compared to the contralateral eyes receiving rAAV-lacZ injection at 5, 10, and 15 days after induction of diabetes.These results are consistent with previous reports that angiostatin reduces the vascular leakage via blockade of VEGF [13-15,39,40]. How angiostatin reduces vascular leakage through inhibition of VEGF production is still under investigation.

It is known that p42/p44 MAP kinase phosphorylation is induced by hypoxia [41]. This phosphorylation is diminished by angiostatin in microvascular endothelial cells [42]. Phosphorylation of p42/p44 MAP kinase promotes VEGF expression by activating its transcription via recruitment of the AP-2/Sp1 (activator protein-2) complex of the VEGF promoter [43]. It is therefore plausible that inhibition of phosphorylation of p42/p44 MAP kinase by angiostatin suppresses VEGF expression under conditions of hypoxia. In our study, retinal phosphorylation of p42/p44 MAP kinase decreased in eyes receiving rAAV-angiostatin at one day after induction of diabetes (Figure 8). Our results in STZ-induced diabetic rats suggest that angiostatin suppresses VEGF expression by inhibition of phosphorylation of the p42/p44 MAP kinase.

BRB breakdown is a hallmark of vascular leakage in diabetic retinopathy and other retinal vascular diseases [44,45]. The tight junctions between the retinal vascular endothelial cells constitute an essential structured component of BRB [18]. This barrier limits diffusion of molecules from vessel lumen to the tissue, and thereby maintains the microenvironment of the retina [46]. The barrier protein occludin is decreased in experimental diabetes [21]. VEGF stimulates phosphorylation and redistribution of occludin [19], which is subsequently endocytosed and degraded [47,48]. This process is closely related to the elevated vascular permeability in experimental diabetes [18]. In our study, retinal occludin content in STZ-induced diabetic rats was preserved in eyes receiving rAAV-angiostatin as compared to eyes receiving rAAV-lacZ. The rAAV-angiostatin-induced inhibition of VEGF may therefore suppress vascular leakage by preserving retinal occludin content in STZ-induced diabetic rats

PEDF is a potent angiogenic inhibitor that is counter balanced by the angiogenic effect of VEGF [49-51]. Decreased expression of PEDF in retina is associated with ischemia-induced retinal neovascularization and proliferative diabetic retinopathy [50]. Recently, another proteolytic fragment of plasminogen-kringle 5 (K5) was noted to upregulate PEDF expression in a dose-dependent manner in vascular endothelial cells and in the retina [15]. In our study, there was no statistically significant difference between PEDF expression in eyes receiving rAAV-angiostatin and the contralateral eyes receiving rAAV-lacZ. Our results also revealed that rAAV-angiostatin did not upregulate PEDF expression in HUVEC-2C cells. However, PEDF expression has been shown to be induced at both the mRNA and protein level following injury in the eye [52]. Further research is warranted to explore the effect of rAAV-angiostatin on PEDF expression.

Our study showed that transgenic expression of rAAV-angiostatin can reduce retinal vascular leakage in STZ-induced diabetic rats. This effect is associated with downregulation of retinal VEGF and phospho-p42/p44 MAP kinase expression, and a reduction in the retinal occludin loss induced by diabetes. However, the vascular leakage and VEGF expression after induction of diabetes in the SD rat model was demonstrated to be a short-term effect [53]. Gene-based therapies can be as effective and viable as real treatment if long-term expression can be achieved. To demonstrate the long-term effect of rAAV-angiostatin on vascular leakage induced by diabetes, an alternative animal model such as the Brown-Norway rat could be used in a future study.

Diabetic macular edema is a major cause of vision loss in diabetic patients [7]. On the basis of these findings, we believe that a similar vector and therapeutic gene could eventually be a useful strategy for long-term preventive or adjunctive therapy for macular edema induced by diabetes. It could serve as the basis for an alternative treatment for patients who are suffering from diabetic macular edema as well as a potentially preventive therapeutic modality for diabetic patients who are at risk for development of macular edema.
